# MicroRNA Response and Toxicity of Potential Pathways in Human Colon Cancer Cells Exposed to Titanium Dioxide Nanoparticles

**DOI:** 10.3390/cancers12051236

**Published:** 2020-05-14

**Authors:** Wen Li, Ming Xi Jia, Jing Deng, Jian Hui Wang, Zavuga Zuberi, Sheng Yang, Jie Ba, Zhu Chen

**Affiliations:** 1 National Engineering Laboratory for Deep Process of Rice and Byproducts, College of Food Science and Engineering, Hunan Province Key Laboratory of Edible Forestry Resources Safety and Processing Utilization, Central South University of Forestry and Technology, Changsha 410004, China; liwen@hut.edu.cn (W.L.); Mingxijia123@163.com (M.X.J.); T20071464@csuft.edu.cn (J.B.); 2 Key Laboratory of Biological Nanomaterials and Devices, College of Packaging and Material Engineering, College of Life Sciences and Chemistry, Hunan University of Technology, Zhuzhou 412007, China; zuberiz@nm-aist.ac.tz (Z.Z.); 13949@hut.edu.cn (Z.C.); 3 School of Chemistry and Food Engineering, Changsha University of Science and Technology, Changsha 410114, China; wangjh0909@csust.edu.cn; 4 Department of Science and Laboratory Technology, Dares Salaam Institute of Technology, Dares Salaam P.O. Box 2958, Tanzania; 5 School of Energy Science and Engineering, Central South University, Changsha 410083, China; ceshyang@csu.edu.cn

**Keywords:** bioinformatics analysis, cytotoxicity, human colon cancer cell, TiO_2_-NPs

## Abstract

Titanium dioxide nanoparticles (TiO_2_-NPs) are widely used for biomedical and food applications, the toxicity of TiO_2_-NPs in vivo and in vitro has been elucidated, but the underlying cytotoxicity of TiO_2_-NPs against microRNA remains largely unknown. The purpose of this study was to analyze microRNA profiling induced by TiO_2_-NPs against NCM460 and HCT116 cell lines. Comparative analysis identified 34 and 24 microRNAs were significantly altered in the TiO_2_-NPs treated cells at concentrations of 3 μg/mL and 30 μg/mL, respectively. Functional classification demonstrated that a large proportion of genes involved in metabolism, human disease, and environmental information process were significantly upregulated by TiO_2_-NPs. Bioinformatics analysis suggested that microRNA 378 might be an early indicator of cellular response to exogenous stimuli with apoptotic signals. Furthermore, TiO_2_-NPs significantly altered the expression of microRNA 378b and 378g in HCT116 and NCM460 cell lines at different concentrations from 3 to 6 μg/mL. These concentrations elicit high-sensitivity of stimuli response in colon cancer cells when exposed to the slight doses of TiO_2_-NPs. Our study indicated that microRNAs 378b and 378g may play an important role in TiO_2_-NPs-mediated colonic cytotoxicity, which may provide a valuable insight into the molecular mechanisms of potential risks in colitis and colon cancer.

## 1. Introduction

Nanomaterials have unique physical, chemical, electronic, optical, and catalytic properties, which are widely used in chemical industry, biomedicine, energy, environment, food production, aerospace, and other important fields [1,2,3]. The increasing use of nanomaterials will inevitably lead to an increase in the exposure of nanomaterials in the ground, resulting in negative effects in the application process, and its potential impacts have gradually attracted people’s attention in human health and environmental complications [4,5,6,7]. In humans, drugs may induce toxicity leading to blood vessel division, causing contaminants or harmful microorganisms to enter into the human tissues or organs due to attachment to the nanomaterials, resulting in significant effects [4,6]. Over the past few decades, the field of nanotoxicity has significantly evolved through the booming of nanotechnology to address potential prolonged exposure of nanoparticles to the consumers through medical applications [5,6].

Titanium dioxide nanoparticles (TiO_2_-NPs) are widely used for biomedical applications, drug research and development design, food additives and packaging materials, and so forth [1,2,4,7,8]. Additionally, TiO_2_-NPs can enter the human body and induce toxicity into different organs including, liver [7,8,9], kidney [10,11], lung [12], brain [7,9,13], heart [14], ovary and testis [15,16,17]. In relation to this, inflammation is one among of the most important toxic mechanisms following TiO_2_-NPs exposure. However, in toxicological researches, oral exposure of TiO_2_-NPs to intestinal tract has not been well studied compared with skin contact or inhalation route due to lack of established global standards for toxicity levels [18].

Most of the TiO_2_-NPs entering the body are metabolized and absorbed by the intestine and their adverse effects on gastrointestinal tract in humans and animals are rarely concerned. Colon cancer and inflammatory bowel disease (IBD) are the most common occurring diseases worldwide which might be contributed by TiO_2_-NPs exposure in the intestines. Therefore, there is an urgent need to confirm the safety of TiO_2_-NPs in human intestines by considering an increasing number of TiO_2_-NPs exposures.

In recent years, microRNAs have attracted extensive attention in toxicology. MicroRNAs have been shown to play an important role in gene regulation of vertebrate genome [19,20,21]. It is predicted that microRNAs can regulate the expression of one-third of human genes [21,22]. Contrary, it has been found that nanoparticles can significantly alter the expression of microRNAs in cells [23,24,25]. Hence, regulation of microRNAs as a key post-transcriptional regulator has attracted much attention in the molecular mechanism of nanoparticles toxicology [23,24]. Additionally, microRNAs are considered as a "buffer" for impedance gene expression variation [26] and they help to maintain the robustness of biological processes [21,26]. In the face of stimuli caused by environmental changes, the regulation of microRNAs can be used to maintain biological functions, such as reinforcement of transcriptional programs and attenuation of aberrant transcripts in living organisms. 

MicroRNAs may play important regulatory roles in gene expression and protein synthesis in biological systems, while TiO_2_-NPs are mainly digested and absorbed by the gastrointestinal tract. However, entrance of TiO_2_-NPs into the cells may lead to changes in miRNAs regulation and possibly body damage, these changes are urgently needed to be confirmed. Additionally, studying the damage and mechanism of TiO_2_-NPs on colon cells is of great significance in the prevention and treatment of colonic inflammation and colon cancer. Therefore, normal human colon cell lines (NCM460) and human colon cancer cells (HCT116) cell lines were used to investigate the effects of TiO_2_-NPs on microRNAs expression and correlating the expression of microRNAs with target genes in colon cells.

## 2. Materials and Methods

### 2.1. TiO_2_ Nanoparticles Characterization

TiO_2_-NPs of 25nm were applied in a concentration range of 3–60 μg/mL (Deke Daojin, Beijing, China). The morphology of nanoparticles was characterized by transmission electron microscopy (TEM) (JEOL Ltd., Tokyo, Japan) in dry form at Xiamen University, and the crystallized form of nanoparticles was characterized by X-ray diffraction (XRD) (BRUKER AXS GMBH, Karlsruhe, Germany).

For the preparation of TiO_2_-NPs exposure medium, powdered NPs were suspended in a serum-free 1640 basal medium solution, sonicated for 30 min before each use, and then added to the cell culture medium at test concentrations.

### 2.2. Cell Culture and Treatment

Human colon cancer cell lines (HCT116) and normal colon cell lines (NCM460) were provided by the College of Food Science and Engineering of the Central South University of Forestry and Technology, Changsha, China. The content of nano-TiO_2_ in food is supposed to be 5–10g/kg according to the National Standard of the People’s Republic of China Food Safety National Standard GB2760-2011 [27]. Fu et al. reported that Sprague Dawley rats were treated by intratracheal instillation with TiO_2_-NPs at doses of 0.5, 4, and 32 mg/kg body weight, micro-TiO_2_ with 32 mg/kg body weight and 0.9% NaCl, respectively [28]. In other related studies, it was found that TiO_2_-NPs (5, 15, or 30 μg/mL) significantly inhibited the viability of rat Sertoli cells in a concentration-dependent manner [29]. Moreover, we found that 3 and 6 ug/mL TiO_2_-NPs concentration can promote cell proliferation in our pre-experimental research. Based on the above considerations, we selected the concentration gradients as follows: 3, 6, 30, and 60 μg/mL. The cells were cultured in RPMI-1640 medium (Invitrogen Gibco, New York, USA) which contains fetal bovine serum (FBS), 1% Penicillin-Streptomycin. The cells were maintained in petri dish 10 cm in diameter at 37 °C and 5% CO_2_. The RPMI-1640 medium was replaced every second day, and cells were passaged when adherence rate was approximately 85 % by washing with D-Hanks Buffered Saline Solution for three times, then with 0.25% Trypsin-EDTA Solution [30]. Microscopically, when cells became round and dispersed, new medium was added to terminate the activity of trypsin, and trypsin was removed by centrifugation. The cells were suspended in fresh medium and subcultured in HCT116 and NCM460 cell lines, TiO_2_-NPs exposures were performed for 24 h. The two types of cell lines were exposed to 0, 3, 6, 15, 30, and 60 μg/mL TiO_2_-NPs to screen for cell viability.

### 2.3. Cell Viability Assays

MTT cell proliferation and cytotoxicity assay kits (Sangon, Shanghai, China) were used to detect colon cell viability after exposure to different concentrations of TiO_2_-NPs. Cells were seeded in 96-well plates at 1 × 10^5^ cells/well. After 12 h, the adherent growth of cells was observed under a microscope about 60%. Then the cells were exposed to 100 mL of different concentrations of TiO_2_-NPs for 24 h. The cells were washed with D-Hanks and cultured for 4 h at 37 ℃ with 10 μL MTT (5 mg/mL). The formazan crystals were solubilised in 100 μL formazan solubilization solution for 10 min and absorbance read at 570 nm (Molecular Devices, San Jose, CA, USA) [31]. The percentage of cell viability changes was calculated and compared with untreated cells.

### 2.4. Determination of Intracellular ROS Production

Intracellular ROS levels were measured using a fluorescent probe 2′,7′-dichlorodihydrofluorescein diacetate (DCFH-DA) according to the manufacturer’s instructions (Beyotime, Shanghai, China) [32]. The cells were inoculated in a 24-well plate with 1 × 10^5^ cells/holes. After 12 h, the adherent growth of cells was observed under a microscope about 60%. The cells were exposed to 0, 3, 6, 15, 30, and 60 μg/mL TiO_2_-NPs for 24 h, respectively. The cells were then washed three times with D-Hanks and placed in a fresh 1640 medium (without FBS) with 5 μM DCFH-DA for 20–30 min. Then, cells were washed with D-Hank. Immediately, the fluorescence of samples was measured at 488 and 525 nm excitation and emission wavelengths in Spectra Max i3x (Molecular Devices, California, USA). Compared to the cells that were not incubated with TiO_2_-NPs, the inducing rate of ROS formation was calculated to increase the fluorescence intensity after exposure to TiO_2_-NPs.

### 2.5. Small RNA Sequencing

HCT116 colon cancer cell lines were treated in triplicate with 0, 3 and 30 μg/mL TiO_2_-NPs for 24 h for microRNA changes. Then, cells were washed 2× with D-Hanks, and the total RNA was extracted by SanPrep Column RNA Extraction Kit (Sangon Biotech, Shanghai, China) by following the manufacturer’s protocol. The extracted RNA samples were then gel purified and 18–30 nt fragment construction library was selected [33]. Two kinds of adapters were respectively ligated to the ends of the small RNA fragments, and the prepared RNA was amplified by reverse transcription-polymerase chain reaction (RT-PCR). The 100–120 bp range PCR product was separated by PAGE electrophoresis, and by-products such as primer dimer were effectively removed. The isolated PCR product was loaded onto the BGISEQ-500 platform for sequencing. Experimental testing and data processing were performed by the Beijing Genomics Institute (BGI).

### 2.6. Prediction of microRNAs Target Genes

The data for microRNA profiling of TiO_2_-NPs-3 μg/mL and 30 μg/mL treated HCT116 cells are reported in this paper. MicroRNAs target mRNA were predicted using RNAhybrid, miRanda, and TargetScan. The final results of the target genes were predicted in combination of the three databases.

### 2.7. Gene Ontology Enrichment Analysis

Gene Ontology (GO) enrichment analysis finds all GO-terms that are significantly enriched in a list of differentially expressing small RNA’s (DESs) target genes, it also finds the genes that correspond to specific biological functions. To perform this analysis, BGI first maps all genes to GO-terms in the database, which calculates the gene numbers for every term. The hypergeometric test is then used to find significantly enriched GO-terms in the input gene list. This test is based on “GO-TermFinder”, using an algorithm developed by BGI to perform GO function analysis. The method used is described as follows:

(1) $$P=1-\sum_{i=0}^{m-1}\frac{\left(\begin{array}{c} M\\ i \end{array}\right)\left(\begin{array}{c} N-M\\ n-i \end{array}\right)}{\left(\begin{array}{c} N\\ n \end{array}\right)}$$

In this algorithm, N is the number of all genes with GO annotation; n is the number of DES of the target gene in N; M is the number of all genes annotated to a particular GO term; m is the number of DES target genes in M.

### 2.8. Pathway Analysis

Pathway-based analysis helps to further understand the biological functions of targeted genes that differentially express microRNAs. KEGG is used for pathway enrichment analysis. This analysis identifies significantly enriched metabolic pathways or signal transduction pathways in differential expressed microRNAs target genes when compared with the whole-genome background. The formula used is the same as that in GO analysis. However, N is the number of all genes with KEGG annotation; n is the number of DESs target genes in N; M is the number of all genes that are annotated to a specific pathway; m is the number of DESs target genes in M. The p-value is corrected by using the Bonferroni method, a corrected *p*-value < 0.05 is taken as a threshold. KEGG terms fulfilling this condition are defined as significantly enriched KEGG terms.

### 2.9. microRNA RT-qPCR

HCT116 cells and NCM460 cells were exposed to different concentrations of TiO_2_-NPs (0, 3, 15, 30, and 60 μg/mL) for 24 h, and the total RNA was extracted by SanPrep Column RNA Extraction Kit (Sangon Biotech, Shanghai, China) by following the manufacturer’s protocol. microRNA RT-PCR was performed by the microRNA first-strand cDNA Synthesis (Stem-loop method) from Sangon Biotech Co., Ltd (Shanghai, China) according to the manufacturer’s instructions. Briefly, 3 μg of total RNA was used to make the first-strand cDNA: 10 μL 2× microRNA L-RT solution mix, 1.5 μL microRNA L-RT enzyme mix, 1μL target gene’s stem-loop primer (10 μM), 1 μL U6 gene’s stem-loop primer (10 μM), 3 μg RNA sample and RNase free water to 20 μL. Gently mixed and centrifuged for 3~5 s. The reaction mixture was warmed at 16 °C for 30 min, then heated at 37 °C for 30 min, and at 85 °C for 5 min to inactivate the enzyme and then stored at 4 °C.

Fluorescence quantitative PCR was performed by using the prepared first strand cDNAs, were 1 μL/sample was used for one PCR reaction (in a 96-well plate) for each microRNA (three replicate wells per sample). Each PCR reaction (20 μL) contained 10 μL of 2× microRNA qPCR master mix, 0.5 μL forward primer (10 μM), 0.5μL of reverse primer (10 μM), 1 μL RT product and RNase-free water to 20 μL. The qPCR was performed with the CFX96 Touch (Bio-Rad, Hercules, CA, USA) and the reaction was set as follows: initial denaturation at 95 °C for 40 s followed by 40 cycles of denaturation at 95 °C for 5 s, annealing at 62 °C for 20 s and extension at 70 °C for 10 s. The primer sequences used are shown in Table 1 and Table 2.

### 2.10. Statistical Analysis

Results of at least three independent experiments are presented as mean ± SD. The comparison between treated and control groups was carried out by ANOVA analysis using the SPSS 23.0 software package (SPSS Company, Chicago, IL, USA). * *p* < 0.05 and ** *p* < 0.01 were considered as significant levels for all the analyses performed.

## 3. Results

### 3.1. Nanomaterials Characterization

**Figure 1:**
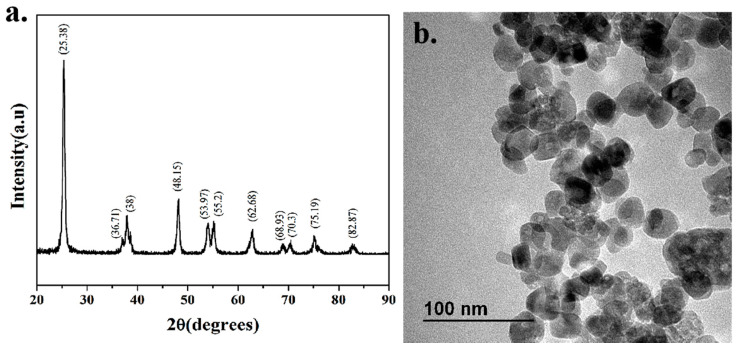
Characterization of nanomaterials. (**a**) XRD pattern of titanium dioxide nanoparticles (TiO_2_-NPs); (**b**) TEM image of TiO_2_-NPs.

The XRD spectra show that the diffraction peaks of powdered TiO_2_-NPs samples are consistent with those of standard anatase, and there are almost no impurity peaks, indicating that the samples have similar properties to anatase and have high purity (Figure 1a). The TEM spectra show that the TiO_2_-NPs are well-dispersed, uniform size, and complete morphology, with an average particle size of about 25 nm (Figure 1b).

### 3.2. Cell Viability

**Figure 2:**
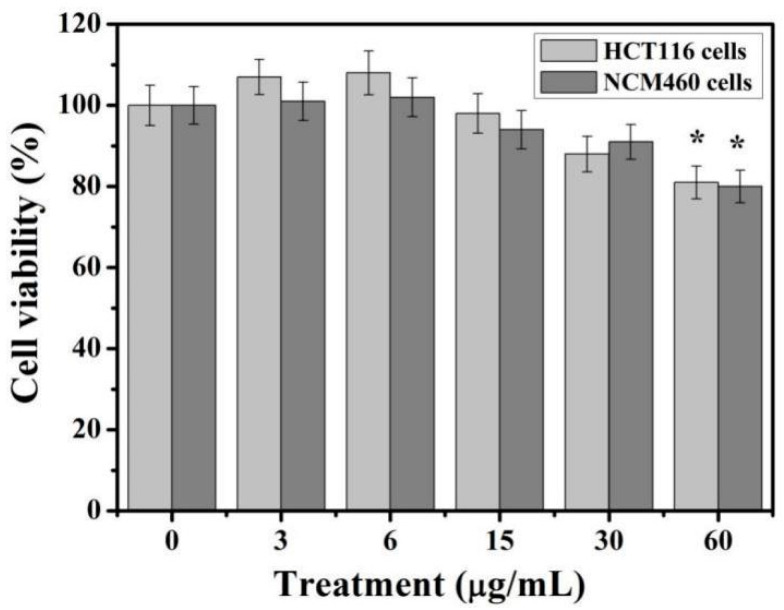
Cytotoxicity of TiO_2_-NPs against HCT116 and NCM460 cell lines. The experiments were carried out in triplicate and data presented are in mean values. * indicates *p* < 0.05.

The two types of cells were treated with TiO_2_-NPs at doses ranging from 3 to 60 μg/mL, respectively. Cell viability was measured by MTT analysis after 24 h of treatment (Figure 2). At a concentration of 3–6 μg/mL, there was no significant change in the activity of the two colon cells. It is worth mentioning that the viability of HCT116 cells increased by about 8–10% (although there were no statistically significant differences, but there was an increasing trend). When the concentration of TiO_2_-NPs reached 60 μg/mL, the viability of both cells decreased to about 80%. TiO_2_-NPs showed slight toxicity to colon cells and inhibited cell viability. In the study of the effects of TiO_2_-NPs on colorectal cancer cells and HUVEC cells, it was also found that TiO_2_-NPs have different effects on the survival rates of these three cell lines [34]. A total of 100 and 400 μg/mL TiO_2_-NPs significantly increased the survival and proliferation of HCT116 cells (62–68%), but not in HT29 cancer cell lines. At the same time, HT29 cells exposed to varied concentrations between 50 and 400 μg/mL TiO_2_-NPs showed approximately 20–30% reduction in viability. The use of higher concentrations of nanoparticles may be related to the different properties of the material. 

### 3.3. Intracellular ROS Analysis 

**Figure 3:**
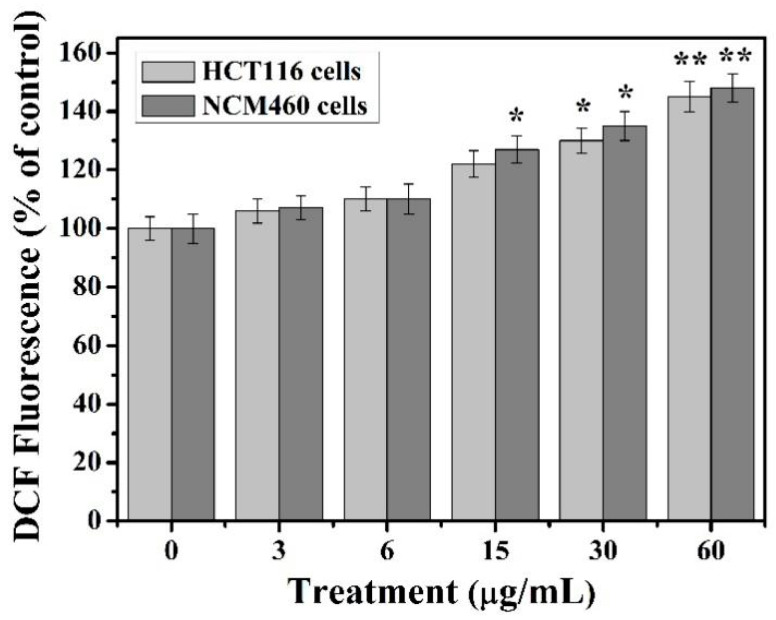
TiO_2_-NPs induces oxidative stress. The cells were treated with 3 to 60 μg/mL of titanium dioxide NPs within 24 h. The experiments were carried out in triplicate and data presented are in mean values, * indicates *p* < 0.05, ** indicates *p* < 0.01.

TiO_2_-NPs increased ROS production in two types of colon cell lines (Figure 3). The two cell lines showed similar results in the analysis of ROS production, and the ROS content of the cells showed a dose-dependent relationship with TiO_2_-NPs. When the concentration of TiO_2_-NPs was 3–6 μg/mL, there was no change in ROS content. When TiO_2_-NPs was 15 μg/mL, the ROS content increased, about 20–25%. As the concentration of nanoparticles increased, the ROS content in both types of cells significantly increased. At higher doses of 30 and 60 μg/mL, the increase in ROS was more pronounced, about 40% and 45%, respectively.

### 3.4. Effects of TiO_2_ NPs on the Expression of microRNAs

Except for Small RNA Sequencing experiments, the other experiments were tested in two cell lines. This is because, through cytotoxicity experiments, we found that 3 μg/mL TiO_2_-NPs may have a potential role in promoting cell viability, while 30 μg/mL TiO_2_ inhibits cell viability. However, this difference was not shown in the NCM460 cell line, indicating that low concentrations of TiO_2_-NPs may promote the growth and inflammation of HCT116 cells. We studied the microRNA changes in HCT116 cells after exposure to 3 and 30 μg/mL TiO_2_-NPs. Except for small RNA sequencing experiments, the other experiments were tested in two cell lines. 

#### 3.4.1. Small RNA Annotation

**Figure 4:**
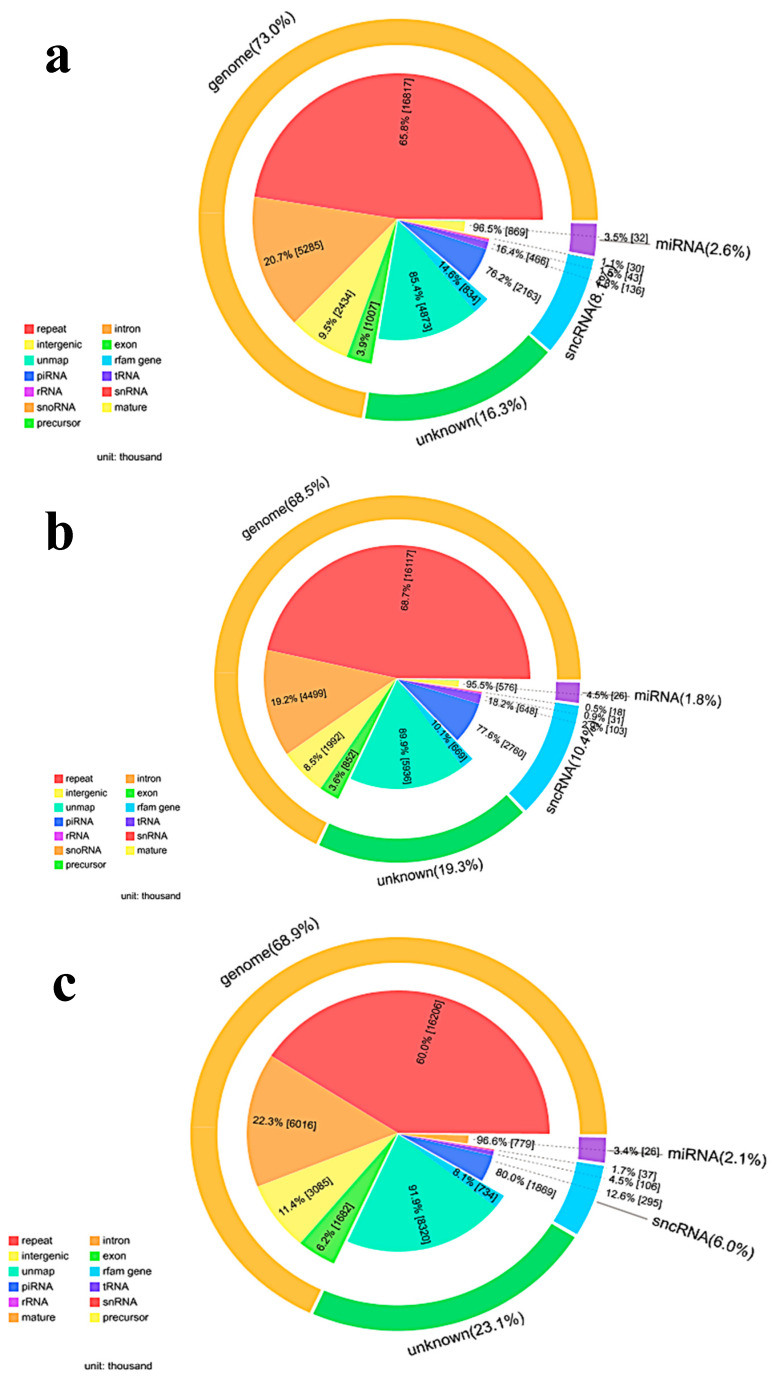
Proportion of small RNAs among different categories. To make every unique small RNA mapped to only one annotation, we follow the following priority rule: microRNA > piRNA > snRNA > Rfam > other RNAs. (**a**) Pie diagram for annotation of small RNA in control HCT116 cell lines (total for each category); (**b**) Pie diagram for annotation of small RNA in TiO_2_-NPs-3μg/mL treated HCT116 cell lines (total for each category); (**c**) Pie diagram for annotation of small RNA in TiO_2_-30 μg/mL treated HCT116 cell lines (total for each category).

After filtering, clean tags were mapped to small RNA database. Table 3 lists separate mapping rate for each sample and Figure 4 shows the distribution of tags. Figure 4a, 4b and 4c show the distribution of unique expressed small RNAs among different categories in control, TiO_2_-NPs-3 μg/mL and 30 μg/mL treated HCT116 cell lines, respectively. The numbers of annotated total microRNAs were most in TiO_2_-NPs-30 μg/mL treated HCT116 cell lines, then in control HCT116 cell lines, and the least in 3 μg/mL treated HCT116 cell lines. However, there were no significant differences in the number of microRNAs labeled in the three sample groups.

#### 3.4.2. Analysis of Significant Differences in microRNAs

**Figure 5:**
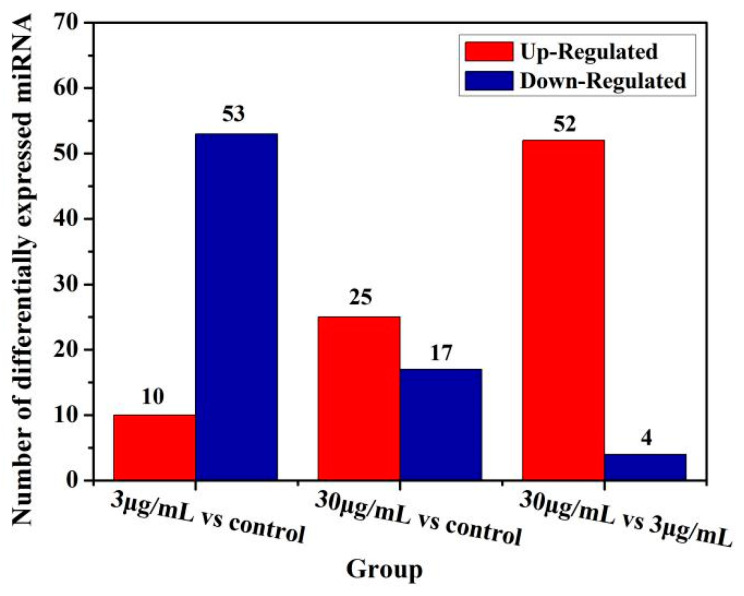
Graph of statistical numbers of differentially expressed microRNA. X-axis represents each pair of differential alignment schemes, and Y-axis represents the corresponding number of differential microRNAs. A vs B means sample B is the control and sample A is the case. If a gene is upregulated it means the expression of this gene is upregulated in sample A compared to sample B.

Compared to the global expression of control cells, 34 and 24 genes expression was significantly altered (*p* < 0.05) after 3 and 30 μg/mL of TiO_2_-NPs treated for 24 h, respectively. In 3 μg/mL TiO_2_-NPs treated cells, 27 genes were downregulated and 7 genes were upregulated, while 13 genes were downregulated and 11 genes were upregulated in 30 μg/mL TiO_2_-NPs treated cells (Figure 5). Table 4 shows some of the differentially expressed microRNAs.

#### 3.4.3. GO Function Analysis

**Figure 6:**
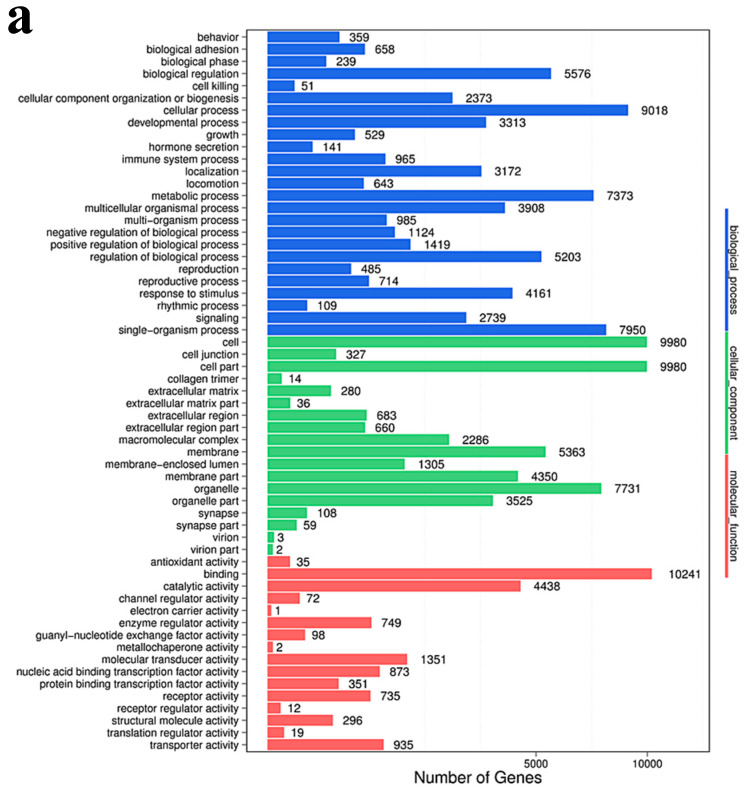

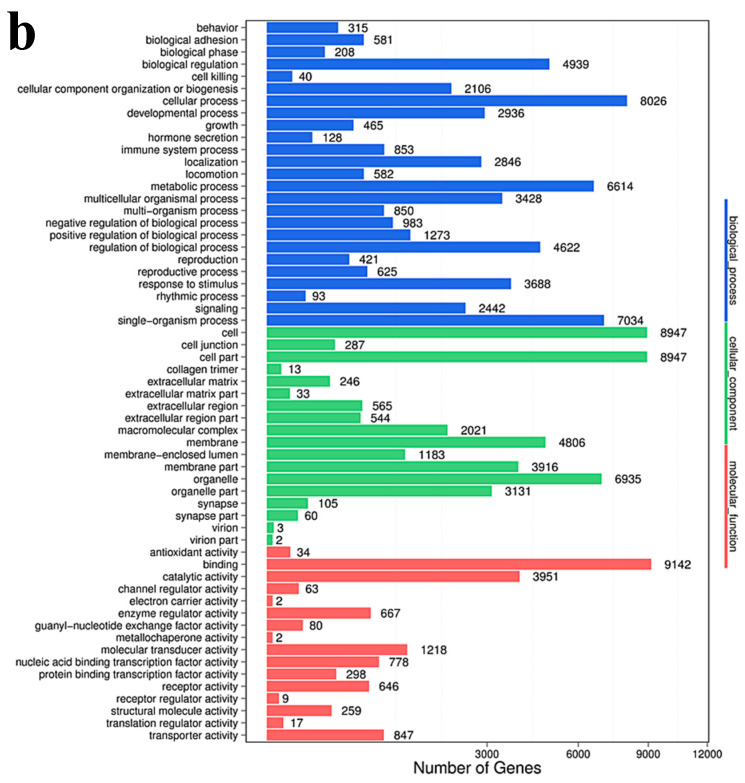
GO classification map of target genes of differential microRNAs. (**a**) 3 μg/mL group VS control group; (**b)**. 30 μg/mL group VS control group. *X*-axis represents number of DEGs (the number is presented by its square root value), *Y*-axis represents GO terms. All GO terms are grouped into three ontologies: blue is for biological processes, brown is for cellular component, and orange is for molecular function.

The target genes of all differential microRNAs were functionally enriched and classified by Gene Ontology, and the functional distribution characteristics of the differential genes were macroscopically recognized. Using all the coding protein genes as background genes, the p-value was calculated using the hypergeometric distribution and *p* < 0.05 was set as the significance threshold value. A high-frequency annotation with statistical significance relative to the background was obtained, so that the distribution information of the gene collection on the GO category can be obtained. GO has three ontologies: molecular function, cellular component, and biological process. The GO function of DESs target genes is mainly concentrated in the biological process, and the GO enrichment results of the DESs target genes of the experimental groups of 3 and 30 μg/mL were basically the same (Figure 6).

#### 3.4.4. KEGG Pathway Analysis

**Figure 7:**
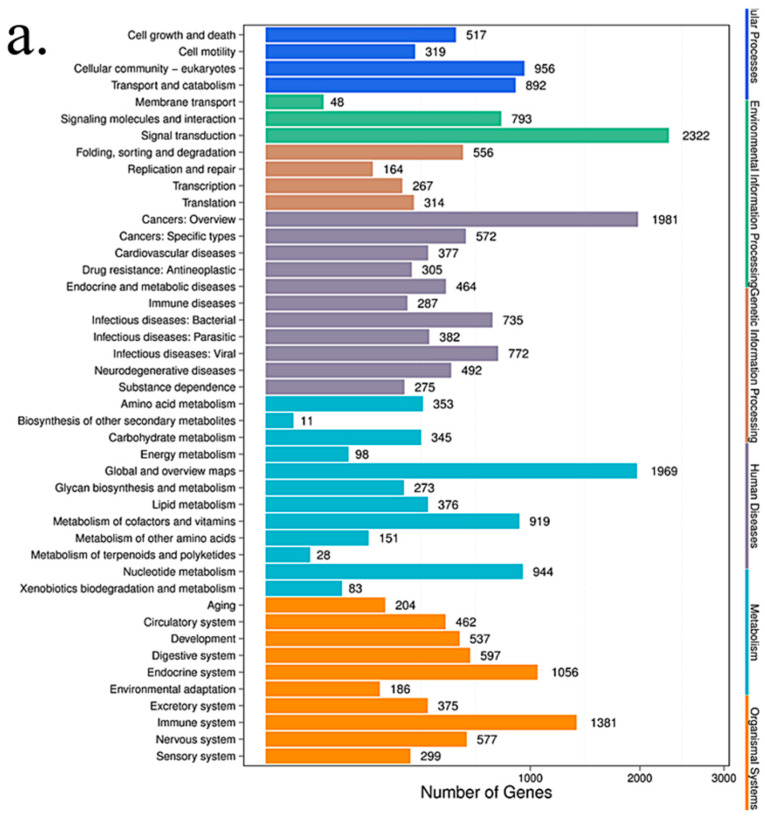

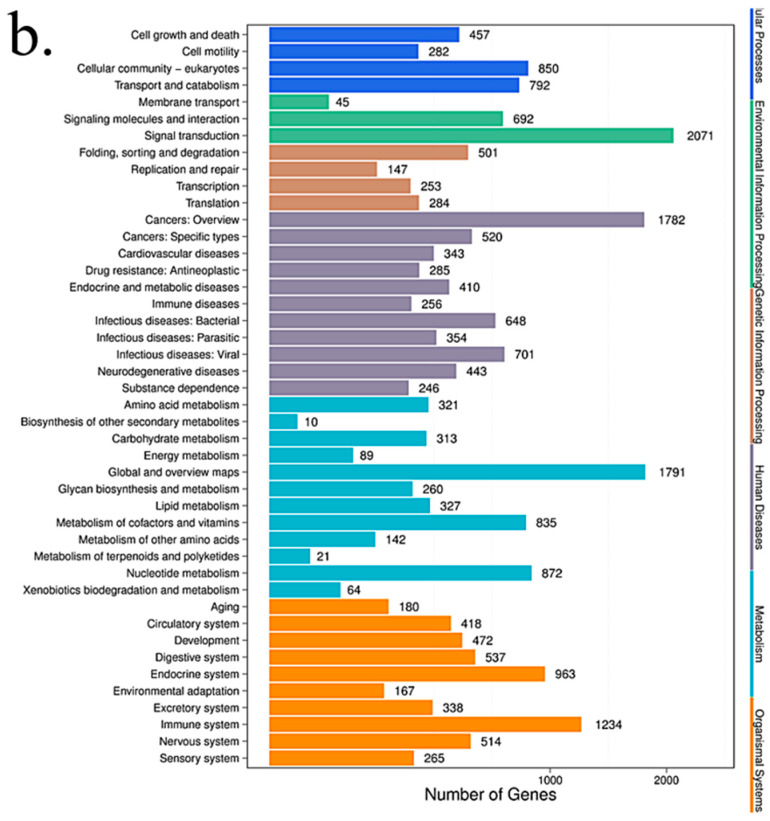
The KEGG pathway annotation classification chart of the differential microRNA target gene. (**a**) 3 μg/mL group VS control group; (**b)** 30 μg/mL group VS control group. The abscissa is the number of target genes that differ in small RNA, and the ordinate represents the secondary KEGG pathway. The secondary pathways belong to different primary pathways, respectively, and different levels represent the first-level communication categories.

We looked for canonical pathways altered by TiO_2_-NPs treatment (Table 5). Since HCT116 is a colon cancer cell line, pathways that occurred in other cell types were not included in this analysis. Under these criteria, there are significant changes in the canonical pathways in TiO_2_-NPs treated HCT116 cells (Figure 7). In these pathways, the enriched pathway can be roughly categorized into 4 different groups. Metabolism (Thiamine metabolism, Purine metabolism, Glycosaminoglycan biosynthesis-chondroitin sulfate/dermatan sulfate, Glycosaminoglycan biosynthesis-heparan sulfate/heparin, Metabolic pathways, Lysine degradation, Retinol metabolism), Human Diseases Cancers (Transcriptional misregulation in cancer, microRNAs in cancer, N-Glycan biosynthesis), Environmental Information Processing (Notch signaling pathway, ECM-receptor interaction, Hedgehog signaling pathway, mTOR signaling Pathway, Phosphatidylinositol signaling system), Cellular Processes (Gap junction, Endocytosis, Signaling pathways regulating pluripotency of stem cells), which may be regulated by corresponding microRNAs. microRNAs may play an important role in TiO_2_-NPs-mediated colonic cytotoxicity.

According to Table 6 and Table 7, it can be found that the target genes of microRNA 378b and 378g are involved in basic biological processes and functions such as cell metabolism, transcriptional regulation, protein modification, cell proliferation, differentiation, and apoptosis. Among the more familiar target genes related to cell growth, apoptosis and metabolism are MAPK1, CASP5, BCL2L2, MAPK3, and so on. In related research, it was found that nano-TiO_2_ significantly inhibited the viability of rat Sertoli cells in a concentration-dependent manner. Additionally, upregulated the expression of p-PKC and p-p38/MAPK in cells in a dose-dependent manner, thereby inducing cellular immune dysfunction, thereby triggering NF-κB activation and eventually inducing the expression of inflammatory factors in cells [35]. The MAPK pathway may be regulated by microRNA 378b and 378g to affect cell viability. The expression levels of miRNA 378b and 378g were verified in two colon cells by RT-PCR for their possible regulatory effects.

### 3.5. RT-qPCR 

**Figure 8:**
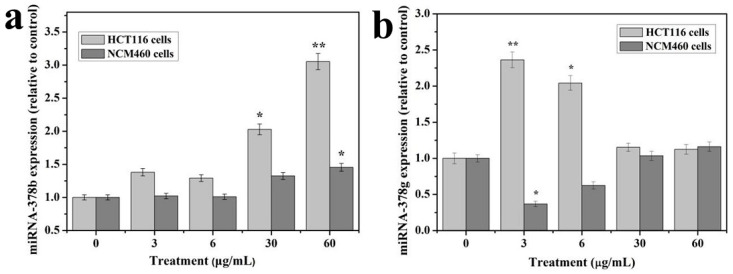
The expression patterns of microRNAs 378b and 378g at 3 and 60 μg/mL treatments in HCT116 and NCM460 cell lines. (**a**) Expression of microRNAs 378b; (**b**) Expression of microRNAs 378g. The experiments were carried out as in triplicate and data presented are in represent mean values. * indicates means *p* < 0.05, ** indicates means *p* < 0.01.

The expression levels showed a dose response for microRNA 378b (Figure 8a) and 378g (Figure 8b) from 3 to 30 μg/mL TiO_2_-NPs treatments. And microRNA 378b were significantly altered. Overall, the data from qRT-PCR correlated well with the microRNA data and demonstrated the changes of these microRNAs at 3 and 30 μg/mL treated samples.

MicroRNAs were extracted from HCT116 and NCM460 cells treated with TiO_2_-NPs and verified the expression changes of microRNA 378b and 378g by qRT-PCR. The results showed that the expression of miRNA 378b and miRNA 378g were consistent with the differential gene expression detected by HCT116 cell microRNA expression profiling, which proved the accuracy of microRNA expression profiling data. The expression of miRNA 378b was stable in NCM460 cells treated with TiO_2_-NPs, and the expression of miRNA 378g was decreased when treated with 3-6 μg/mL TiO_2_-NPs, and the expression increased with the increase of TiO_2_-NPs concentration. When the concentration was increased to 30-60 μg/mL, the expression level returned to normal. The differential expression of miRNA 378b and 378g induced by TiO_2_-NPs may be related to the cell line type. Our research group will conduct further studies to assess the regulatory effects of the differential expression of miRNA 378b and 378g in the two colon cell lines on related target genes. 

## 4. Discussion

Toxicological effects of nanoparticles are to reveal the basic laws of interaction between nanoparticles and living systems, which ensure the steady development of nanotechnology itself [3,4]. The TiO_2_-NPs can easily enter the cell through the cell membrane because of the ultra-fine nature [13,14]. However, due to its large-specific surface area and high-reactivity, it may interact with many organelles and biological macromolecules in the cell, which can cause the intracellular microenvironment and cell function changes, and even cause genetic mutations and gene-level damage [12,13,14,15,16,17]. But so far, the mechanism of nanoparticle-induced tissue and cell damage is unclear, and the molecular level changes involved in this process are still unclear. Nevertheless, most researches have been focusing on the toxicity mechanism, exposure effects, toxicity effects, migration and transformation pathways, and risk prediction of TiO_2_-NPs [5,6,9,16,17]. 

Recently, studies on the interaction of biological macromolecules and molecular regulation mechanisms has just begun [5,17,19]. At present, studies on various cell lines have shown that TiO_2_ nanoparticles (NPs) induced the inflammatory response and cytotoxicity [36,37]. However, little is known about the potential mechanism of microRNA response in colon cells caused by TiO_2_-NPs exposure. In this study, HCT116 and NCM460 cell lines were selected to evaluate the colonic cytotoxicity of TiO_2_-NPs. Similarly, cell viability testing is a vital step in toxicology testing which shows the response of cells to drugs and provides information about cell death and survival [5,13,14,17,18,19,36,37]. In our study, when the concentration of TiO_2_-NPs reached 60 μg/mL, the viability of both cells decreased to about 80%. These results indicate that TiO_2_-NPs are slight toxic to colon cells and inhibited cell viability. 

The ROS mechanism is one of the most commonly accepted oxides for assessing the toxicity of nanomaterials [9,14]. Similarly, nanoparticles with active ingredients on the surface enhance their ability to produce ROS [5,6]. ROS mainly include superoxide ions, hydrogen peroxide, hydroxyl radicals, etc. Trace reactive oxygen species play an important role in the regulation of certain physiological phenomena, while excess reactive oxygen species can cause oxidative damage to cellular macromolecules [37,38]. If exogenous substances cause overactive oxygen in the body, or reduce the amount of antioxidants, the structure and function of cells may be destroyed when the balance of ROS in the body is destroyed [38], and oxidative damage also causes apoptosis [38,39]. Our results show that the content of reactive oxygen species in HCT116 and NCM460 cells increased with the increase of TiO_2_-NPs concentration under the treatment of less than 60 μg/ml. Moreover, the intracellular ROS content considerably increased compared to control group at the concentration of 30–60 μg/mL TiO_2_-NPs and reached the highest at 60 μg/mL concentration. This indicates that the excessive accumulation of ROS induced by TiO_2_-NPs may be an important cause of cytotoxicity.

Due to unique physical and chemical properties of nanomaterials, traditional toxicological detection methods are not often completely applicable. Especially under low-dose exposure of nanomaterials (such as environmental exposure conditions), there is no significant oxidative stress damage, some indirect toxic effects such as secondary toxic reactions and compensatory damage effects are often ignored [39]. In the maintenance and transmission of epigenetic information, RNA editing is an important carrier. RNA levels can change rapidly to alter the stress and metabolic state of cells under endogenous and exogenous stimuli [23,24,40,41,42]. Therefore, microRNA expression levels are considered as sensitive early indicators of the cell/ body response to endogenous and exogenous stimuli [23,24,42]. 

At present, there are many unknowns about the changing rules and related mechanisms of the changes in microRNA levels caused by nanomaterial exposures [43,44]. There are many regulatory factors in the development and progression of cancer. However, in vivo studies have demonstrated that titanium dioxide facilitates growth of chemically induced colorectal tumors and induce transcriptomic changes suggestive of an impaired immune system and cancer development [35,36,43,44]. MicroRNA regulates cell proliferation, differentiation, and apoptosis, such as cytokines, transcription factors, growth factors, pro-apoptotic and anti-apoptotic genes, by regulating the expression of signaling molecules, indicating that microRNAs are closely related to tumorigenesis development [40,41]. Deregulation of microRNA expression may be an important factor in tumorigenesis and cancer progression [42,45]. New researches suggest that certain expression changes in oncogenes or tumor suppressor genes may also lead to colon cancer [43,44,46]. Moreover, *in vitro* studies showed that TiO_2_-NPs induced oxidative stress responses, DNA damage, and micronuclei formation, which showed that TiO_2_-NPs induce gene expression changes related to signaling, the olfactory/GPCR receptor family, oxidative stress, inflammation, immune system, transport and cancer. The gene expression changes associated with the immune system and inflammation induced by TiO_2_-NPs suggest the creation of a favorable environment for colon cancer development [37,43,44]. 

Furthermore, using nuclear factor (NF)-kappa B reporter gene assays, TiO_2_-NPs-induced IL8 mRNA expression occurs in part through activation of NF-kappa B and p38 mitogen-activated protein kinase pathways in the human colon adenocarcinoma cell line Caco-2 [36] and those results demonstrate that TiO_2_-NPs cause an activation of inflammatory pathways in the human colon adenocarcinoma cell line Caco-2. Nevertheless, studying changes in microRNA expression may help to more clearly understand the mechanisms of colon cancer development, progression, migration, and recurrence, as well as contribute to the early diagnosis and treatment of colon cancer. The detailed function of microRNA in colon cancer development has not been clearly defined. However, it has been found that the target pathways of microRNAs in colon tissue include cAMP, Wnt, TGF-β, MAPK, and mTOR signaling pathways [47,48,49]. MicroRNAs may control genes in these pathways to regulate colon tissue development and pathology [50]. 

Moreover, function of some microRNAs has been extensively studied. Although the test dose of TiO_2_-NPs was not significantly cytotoxic to HCT116 cells, the expression of microRNA changed significantly. The expression of microRNA changes was examined in GO function analysis and ingenuity pathway analysis to delineate associated canonical/signaling pathways. The purpose was to analyze microRNA profiling of NCM460 and HCT116 cells after exposure to TiO_2_-NPs. Comparative analysis identified 34 and 24 microRNAs were significantly altered in the TiO_2_-NPs treated cells at concentrations of 3 and 30 μg/mL, respectively. Additionally, functional classification demonstrated that a large proportion of genes involved in thiamine metabolism, purine metabolism, glycosaminoglycan biosynthesis—chondroitin sulfate/dermatan sulfate, metabolic pathways, notch signaling pathway, glycosaminoglycan biosynthesis—heparan sulfate/heparin, transcriptional misregulation in cancer, lysine degradation, ECM-receptor interaction, amoebiasis, cAMP pathway and mTOR signaling pathway were significantly upregulated by TiO_2_-NPs. 

In addition, bioinformatics analysis suggested that microRNA 378 may be an early indicator of cellular response to exogenous stimuli with apoptosis signals. Contrary, many genes are known to be regulated by microRNAs, and the expression of microRNAs is altered by nano-titanium dioxide treatment in these pathways. Hence, the microRNA 378 family that is involved in metabolism, transcriptional regulation, protein modification, cell proliferation, differentiation, and apoptosis were altered by TiO_2_-NPs treatments. However, studies have shown that microRNA 378 has biological functions that regulate a variety of tumor cells, including cell proliferation, migration and invasion, and drug resistance [51,52,53,54]. In gastric cancer, microRNA 378 affects cell proliferation and cell cycle by targeting CDK6 and VEGF genes [52]. Also, microRNA 378 inhibits the growth and proliferation of tumor cells in hepatocellular carcinoma [54]. MicroRNA 378 inhibits proliferation and migration of colon cancer cells by targeting SDAD1 [55]. Specifically, TiO_2_-NPs altered the expression of certain microRNAs and also regulate relevant microRNAs to their target genes [56,57]. This suggests that nanoparticles may disrupt the expression of genes in cells, and as the regulatory systems, microRNAs may correct perturbations by enhancing transcriptional programs or attenuating aberrant transcripts.

## 5. Conclusions

MicroRNAs play a crucial role in our biological systems and this is the first study to investigate toxicological effects of TiO_2_-NPs on the expression of microRNAs in colonic cell lines. MicroRNAs or genes altered by NPs may have adverse effects on the intestinal system by altering certain specific signaling pathways. Altogether, this study indicates that microRNAs may play an important role in TiO_2_-NPs-mediated colonic cytotoxicity, which may provide a valuable insight into the molecular mechanisms of potential risks in colitis and colon cancer. It is, therefore, necessary to conduct further studies in understanding the molecular mechanisms induced by TiO_2_-NPs in the regulation of specific microRNAs.

## Figures and Tables

**Table 1:** List of microRNA reverse transcriptional stem-loop primer sequences.

Gene Name	Loop-RT Primer Sequence
Homo U6	GTCGTATCCAGTGCAGGGTCCGAGGTATTCGCACTGGATACGACAAAATA
hsa-miR-378b	GTCGTATCCAGTGCAGGGTCCGAGGTATTCGCACTGGATACGACTTCTGC
hsa-miR-378g	GTCGTATCCAGTGCAGGGTCCGAGGTATTCGCACTGGATACGACCTTCTG

**Table 2:** List of upstream and downstream primer sequences.

Primer Name	Sequence (5′–3′)
Homo U6 Forward primer	AGAGAAGATTAGCATGGCCCCTG
hsa-miR-378b Forward primer	GCGCGACTGGACTTGGAG
hsa-miR-378g Forward primer	CGCGACTGGGCTTGGAGT
General Reverse primer	AGTGCAGGGTCCGAGGTATT

**Table 3:** Alignment statistics of tags align to reference genome.

Sample Name	Total Tag	Mapped Tag	Percentage (%)
control	34,995,302	29,921,249	85.50
TiO_2_-NPs-3 μg/mL	34,231,512	28,095,346	82.07
TiO_2_-NPs-30 μg/mL	39,190,347	30,681,926	78.29

**Table 4:** List of total microRNAs significantly regulated by TiO_2_-NPs. The fold change was the log2-Ratio value, cut-off at log2-Ratio ≥ 1 or log2Ratio ≤ −1.

TiO_2_-NPs-3μg/mL	Fold Change	TiO_2_-NPs-30μg/mL	Fold Change
hsa-miR-378f	3.520946008	hsa-miR-378e	4.722807531
hsa-miR-103a-3p	2.953306765	hsa-miR-199b-3p	3.924812504
hsa-miR-27b-5p	2.214124805	hsa-miR-4443	2.025090981
hsa-miR-4443	2.201674124	hsa-miR-101-3p	1.893084796
hsa-miR-378g	2.175707743	hsa-miR-1307-5p	1.620411337
hsa-miR-374c-3p	1.415037499	hsa-miR-378b	1.61172941
hsa-miR-378i	−4.07681560	hsa-miR-103a-3p	1.463602466
hsa-miR-374b-5p	−3.824842349	hsa-miR-210-3p	1.429585808
hsa-miR-4284	−3.148259549	hsa-miR-193b-3p	1.180827839
hsa-let-7f-2-3p	−2.626782676	hsa-miR-629-5p	1.152003093
hsa-miR-501-5p	−2.163975735	hsa-miR-339-3p	1.025943267
hsa-miR-24-3p	−2.138268765	hsa-miR-199a-3p	−7.105035471
hsa-miR-3656	−2.080703096	hsa-miR-378i	−6.169925001
hsa-miR-3074-5p	−1.826741314	hsa-miR-378g	−3.798524718
hsa-miR-132-3p	−1.712341807	hsa-miR-548ae-5p	−3.321928095
hsa-miR-339-5p	−1.656663966	hsa-miR-374b-5p	−3.228198043
hsa-miR-3960	−1.506687086	hsa-miR-4284	−1.982249598
hsa-miR-6821-5p	−1.436099115	hsa-miR-132-3p	−1.712341807
hsa-miR-7704	−1.395592382	hsa-miR-1246	−1.695664562
hsa-miR-143-3p	−1.358520148	hsa-miR-339-5p	−1.5334322
hsa-let-7a-3p	−1.311523999	hsa-miR-24-3p	−1.389680675
hsa-miR-135b-5p	−1.300942024	hsa-miR-1290	−1.253756592
hsa-miR-5701	−1.247461602	hsa-miR-143-3p	−1.130466392
hsa-miR-148b-3p	−1.235501962	hsa-miR-5701	−1.003732719
hsa-miR-1290	−1.208769137		
hsa-miR-29b-3p	−1.198023321		
hsa-miR-96-5p	−1.168774188		
hsa-miR-103b	−1.141661149		
hsa-miR-128-3p	−1.132807408		
hsa-miR-4532	−1.114263111		
hsa-let-7i-3p	−1.101879614		
hsa-miR-33b-3p	−1.091524104		
hsa-miR-151b	−1.032454631		

**Table 5:** Altered canonical pathways by TiO_2_-NPs in HCT116 cells.

TiO_2_-NPs-3 μg/mL	TiO_2_-NPs-30 μg/mL
Thiamine metabolism	Thiamine metabolism
Purine metabolism	Purine metabolism
Glycosaminoglycan biosynthesis—chondroitin sulfate/dermatan sulfate	Transcriptional misregulation in cancer
Glycosaminoglycan biosynthesis—heparan sulfate/heparin	Metabolic pathways
Transcriptional misregulation in cancer	Glycosaminoglycan biosynthesis—chondroitin sulfate/dermatan sulfate
Metabolic pathways	Glycosaminoglycan biosynthesis—heparan sulfate/heparin
Notch signaling pathway	Phosphatidylinositol signaling system
ECM-receptor interaction	Notch signaling pathway
Vibrio cholerae infection	Endocytosis
Lysine degradation	Breast cancer
Amoebiasis	Lysine degradation
Hedgehog signaling pathway	N-Glycan biosynthesis
Axon guidance	Hippo signaling pathway—multiple species
cAMP pathway	Signaling pathways regulating pluripotency of stem cells
Gap junction	cAMP pathway
	MicroRNAs in cancer
	Prion diseases
	mTOR signaling pathway
	MAPK/ERK pathway

**Table 6:** List of predicted microRNA 378b target gene.

Target Gene	Gene Name	Cumulative Weighted Context++ Score
GOLT1A	golgi transport 1A	−0.59
SBDS	Shwachman–Bodian–Diamond syndrome	−0.47
EIF4G3	eukaryotic translation initiation factor 4 gamma, 3	−0.47
KIAA1522	KIAA1522	−0.46
VANGL1	VANGL planar cell polarity protein 1	−0.46
ZFP36L2	ZFP36 ring finger protein-like 2	−0.39
PTPRT	protein tyrosine phosphatase, receptor type, T	−0.36
TOB2	transducer of ERBB2, 2	−0.34
ATG12	autophagy related 12	−0.32
MAPK1	mitogen-activated protein kinase 1	−0.31
DACT1	dishevelled-binding antagonist of beta-catenin 1	−0.31
KIF2A	kinesin heavy chain member 2A	−0.31
VAT1L	vesicle amine transport 1-like	−0.29
RBMS1	RNA binding motif, single-stranded interacting protein 1	−0.29
SAMD1	sterile alpha motif domain containing 1	−0.27
SCAMP2	secretory carrier membrane protein 2	−0.27
RNF144B	ring finger protein 144B	−0.26
PIWIL2	piwi-like RNA-mediated gene silencing 2	−0.25
ZDHHC9	zinc finger, DHHC-type containing 9	−0.22
SUFU	suppressor of fused homolog (Drosophila)	−0.22
GSTM4	glutathione S-transferase mu 4	−0.20
CSNK1G2	casein kinase 1, gamma 2	−0.20
UBQLN4	ubiquilin 4	−0.19
ALPK3	alpha-kinase 3	−0.18
CTDP1	CTD (carboxy-terminal domain, RNA polymerase II, polypeptide A) phosphatase, subunit 1	−0.17
DYNC1LI2	dynein, cytoplasmic 1, light intermediate chain 2	−0.17
WDR37	WD repeat domain 37	−0.17
IGF1R	insulin-like growth factor 1 receptor	−0.16
ZKSCAN1	zinc finger with KRAB and SCAN domains 1	−0.15
CADM4	cell adhesion molecule 4	−0.15
FCRLB	Fc receptor-like B	−0.15
CUEDC1	CUE domain containing 1	−0.14
PHF15	PHD finger protein 15	−0.14

**Table 7:** List of predicted microRNA 378g target gene.

Target Gene	Gene Name	Cumulative Weighted Context++ Score
STARD9	StAR-related lipid transfer (START) domain containing 9	−1.01
AKAP13	A kinase (PRKA) anchor protein 13	−0.88
TMEM91	transmembrane protein 91	−0.88
ADM2	adrenomedullin 2	−0.88
HMHB1	histocompatibility (minor) HB-1	−0.84
MYL12B	myosin, light chain 12B, regulatory	−0.79
GP9	glycoprotein IX (platelet)	−0.78
NDUFA4L2	NADH dehydrogenase (ubiquinone) 1 alpha subcomplex, 4-like 2	−0.78
GRAPL	GRB2-related adaptor protein-like	−0.78
DAD1	defender against cell death 1	−0.75
SLC39A4	solute carrier family 39 (zinc transporter), member 4	−0.75
GAS8	growth arrest-specific 8	−0.73
SCAMP5	secretory carrier membrane protein 5	−0.72
ERI3	ERI1 exoribonuclease family member 3	−0.72
CARD16	caspase recruitment domain family, member 16	−0.71
GLTPD1	glycolipid transfer protein domain containing 1	−0.69
IFI30	Interferon, gamma-inducible protein 30	−0.69
CASP5	caspase 5, apoptosis-related cysteine peptidase	−0.68
ME3	malic enzyme 3, NADP(+)-dependent, mitochondrial	−0.64
KCNB1	potassium voltage-gated channel, Shab-related subfamily, member 1	−0.64
PPP4R1L	protein phosphatase 4, regulatory subunit 1-like	−0.64
BCL2L2	BCL2-like 2	−0.56
NKAIN1	Na^+^/K^+^ transporting ATP ase interacting 1	−0.55
MAP3K3	mitogen-activated protein kinase kinase kinase 3	−0.49
PRKACG	protein kinase, cAMP-dependent, catalytic, gamma	−0.47
BOC	BOC cell adhesion-associated, oncogene regulated	−0.47
PRKACA	protein kinase, cAMP-dependent, catalytic, alpha	−0.45
MAPK3	mitogen-activated protein kinase 3	−0.42
GRB2	growth factor receptor-bound protein 2	−0.40
